# An Integrated Deep Network for Cancer Survival Prediction Using Omics Data

**DOI:** 10.3389/fdata.2021.568352

**Published:** 2021-07-16

**Authors:** Hamid Reza Hassanzadeh, May D. Wang

**Affiliations:** ^1^ School of Interactive Computing, Georgia Institute of Technology, Atlanta, GA, United States; ^2^ Department of Biomedical Engineering, Georgia Institute of Technology and Emory University, Atlanta, GA, United States

**Keywords:** deep belief networks, integrated cancer survival analysis, RNA-seq, precision medicine, deep learning, multi-omics

## Abstract

As a highly sophisticated disease that humanity faces, cancer is known to be associated with dysregulation of cellular mechanisms in different levels, which demands novel paradigms to capture informative features from different omics modalities in an integrated way. Successful stratification of patients with respect to their molecular profiles is a key step in precision medicine and in tailoring personalized treatment for critically ill patients. In this article, we use an integrated deep belief network to differentiate high-risk cancer patients from the low-risk ones in terms of the overall survival. Our study analyzes RNA, miRNA, and methylation molecular data modalities from both labeled and unlabeled samples to predict cancer survival and subsequently to provide risk stratification. To assess the robustness of our novel integrative analytics, we utilize datasets of three cancer types with 836 patients and show that our approach outperforms the most successful supervised and semi-supervised classification techniques applied to the same cancer prediction problems. In addition, despite the preconception that deep learning techniques require large size datasets for proper training, we have illustrated that our model can achieve better results for moderately sized cancer datasets.

## Introduction

Advances in big data and high-throughput technologies during the past decade have led to massive accumulation of high-dimensional omics data, which enables the data-driven prediction of disease prognosis using molecular profiles. However, this data-driven prognosis remains challenging because of the interplay of mostly unknown molecular factors from a haystack of millions of molecular features. The general practice in prognosis of most of the malign diseases has been based on the traditional methods without a comprehensive analysis of genetic and molecular profiles. This is primarily due to the lack of reliable clinical decision support systems (CDSSs) that can efficiently model and integrate information into actionable knowledge.

The association of molecular profiles with the onset of chronic diseases and their sub-types and prognoses has been extensively reviewed and reported during the past years (Hsieh et al., 2018; Collisson et al., 2019; Sicklick et al., 2019). Despite the success of a number of these approaches, majority of them utilize the so-called shallow-learners, which often fall short in learning higher-order abstract representations of the data and fail to capture complex inter-modality or intra-modality interactions of features or their relationship with respect to clinical endpoints of interest. Often, shallow learners use a limited set of features derived from the expert knowledge or feature reduction techniques, such as the principal component analysis (PCA). Thus, they are limited in their ability to learn non-linear higher-level informative features. In contrast, deep learning (LeCun et al., 2015) is revolutionizing the field of feature learning (also known as representation learning) in biomedicine (Alipanahi et al., 2015; Fan et al., 2015; Park and Kellis, 2015; Spencer et al., 2015; Wang S. et al., 2016). Inspired by neuroscience, the power of deep learning is its ability to represent high-dimensional data by multiple levels of non-linearity abstractions and to train DL models with more effective optimizations and regularization techniques. Once such a representation is derived, any classifier for the prediction task can use it.

To date, some studies have designed deep methods for prediction and prognosis of cancer using different types of modalities. Fakoor et al. (2020) used a stack of sparse auto-encoders along with an augmenting dimensionality reduction step using PCA, to learn features from gene expression data that can help classify cancer types. They developed three variants of their proposed paradigm and showed that they perform reasonably well across different datasets in some of their devised experiments, but not all. The addition of PCA to extract new features from randomly selected probes is a necessary step in their pipeline as the sparse stacked auto-encoder is not enough by itself to predict informative features. Moreover, their approach uses only a single data modality, i.e., gene expression data, for prediction of cancer type. In another study, Kumar et al. (2015) used a similar approach to Fakoor et al. (2020), in their own domain of interest, to create useful features from CT images to classify benign vs. malignant lung nodules. They showed that their approach resulted in a performance boost compared to the state-of-the-art approaches. Azizi (2020) developed a supervised pipeline, based on the deep belief network (DBN), for detection of prostate cancer given ultrasound temporal data. The author used deep belief networks to learn useful features, which are then fed into a support vector machine classifier to predict cancer. In another study, Liang et al. (2015) integrated several restricted Boltzmann machines (RBMs) for an unsupervised task of grouping cancer tumors into different clusters using cross-platform but same-type molecular data. They showed that patients grouped in different clusters exhibit differentiable Kaplan–Meier survival curves, which is an indication of the soundness of their proposed clustering approach. More recently, Zeng et al. (2020) used a supervised learning approach based on the convolutional neural network for subtyping of breast cancer. Besides the supervised nature of the proposed model there, CNNs are severely restricted in capturing long distance relations, due to their short receptive fields, especially when the number of input features is orders of magnitude larger than the utilized kernel width.

Despite many DL applications in different biomedical areas, their success in cancer prediction and prognosis is still limited. This is because deep architectures require high volumes of labeled data samples (due to their expressiveness, Hastie et al., 2009) to train DL models without data overfitting, which is a requirement not always met in cancer-related domains. In this study, we develop an integrated semi-supervised deep learning for risk prediction in cancer cohorts with patients’ molecular profiles. We present an integrated deep architecture to predict cancer survival given the molecular profiles of cancer tumors. We show that our integrated deep model can leverage the available unlabeled data to enhance learning our deep model, a task that is often achieved using semi-supervised learning frameworks. Furthermore, we illustrate that the proposed pipeline outperforms the support vector machine (SVM), a supervised learner that has been successfully used in cancer-related domains (Kim et al., 2012a; Ahmad, 2013; Tseng et al., 2014) as well as the Laplacian SVM, an important graph-based semi-supervised learning paradigm that is promising in solving similar problems (Kim et al., 2012b; Kim and Shin, 2013; Park et al., 2013).

## Datasets

The Cancer Genome Atlas (TCGA) (Weinstein et al., 2013) data portal in the NCI/NIH (Cerami et al., 2012; Wang Z. et al., 2016) hosts multi-modality data of thousands of patients. In this study, we used data from kidney renal clear cell carcinoma (KIRC) and head and neck squamous cell carcinoma (HNSC) diseases from the TCGA data bank. We selected KIRC and HNSC because they are moderately sized. On the one hand, they are not too small^1^ in the number of specimens profiled, and on the other hand, we did not intend to select a cancer type with a relatively large number of samples, such as the invasive breast carcinoma, to showcase the efficacy of the utilized architecture in learning generalizable models. Furthermore, we downloaded RNA-seq expression profiles of patients suffering from neuroblastoma (NB) pediatric cancer from a previously published study (Zhang et al., 2015). For the RNA-seq expression profiles, we used three data modalities per sample, namely, the gene, the isoform, and the junction. For the KIRC and HNSC datasets, these were produced by Illumina HiSeq 2000 platforms and quantified by RSEM (Li and Dewey, 2011). In case of the NB dataset, we selected the results of mapping the reads to the AceView (Thierry-Mieg and Thierry-Mieg, 2006) annotation through the Magic alignment tool (Thierry-Mieg and Thierry-Mieg, 2006). We also used the miRNA expression profiles for the KIRC and HNSC datasets, which were generated by the Illumina GAIIx platform, and finally, the Illumina Infinium HumanMethylation27 platform produced the DNA methylation data for the KIRC disease only. Table 1 lists the available modalities and their statistics for each dataset.

**TABLE 1:** Data description.

Data modality (platform)		Dataset	# of features	# of available samples		
				Labeled		Unlabeled
				Pos.	Neg.	
RNA-seq (Illumina HiSeq 2000)	Gene	KIRC	20533	110	141	281
		HNSC	20533	115	128	276
		NB	60780	115	104	279
	Isoform	KIRC	73601	110	141	281
		HNSC	73601	115	128	276
		NB	263546	115	104	279
	Junction	KIRC	249579	110	141	281
		HNSC	249579	115	128	276
		NB	340416	115	104	279
miRNA (Illumina GAIIx 2000)		KIRC	1048	106	150	269
		HNSC	1048	116	130	276
Methylation (Illumina Infinium HumanMethylation27)		KIRC	21403	111	142	520

## Materials and Methods

Recent years have witnessed a surge of interest in deep learning (DL) and its successful applications in different domains (see promising examples in Hassanzadeh and Wang 2016; Esteva et al., 2017; Hassanzadeh et al., 2017; Miotto et al., 2017), such as image processing, speech recognition, computer vision, and more recently in biology. Despite its success in a wide range of areas, there are only a handful of studies reporting success stories about the application of DL in cancer-related domains. In fact, several attempts to deploy DL in biomedical domains have failed to outperform other classical methods (Fakoor et al., 2020; Ditzler et al., 2015). This is due to the selection of wrong components and the DL architectures for the selected tasks. Moreover, these pipelines are often designed for supervised tasks, which are inefficient when dealing with censored data that are prevalent in cancer databases. In this study, we developed a deep learning model to deal with the dataset size limitation. This strategy is equivalent to the semi-supervised learning (SSL) strategy, where we leverage unlabeled samples to guide the training process of network weights. Until recently, semi-supervised learning (SSL) (Chapelle et al., 2009) approaches have been the dominant practice to learn models that use both labeled and unlabeled data. This is mainly due to their higher performance compared to the purely supervised or unsupervised techniques. Different SSL paradigms try to take advantage of unlabeled data in different ways, but they all capture the probability distribution of the input samples either directly or indirectly. In other words, what gives SSL techniques an advantage over the supervised methods is their ability to exploit all data, irrespective of the labels, to model a more realistic marginal distribution of the input.

### Data Description

Among all SSL techniques, Laplacian support vector machine (LapSVM) is an outstanding recent technique that falls under the category of graph-based SSL paradigms, which builds a graph representation of the data (labeled and unlabeled) based on domain knowledge or the similarity among samples. It has shown the state-of-the-art performance in semi-supervised classification problems (Belkin et al., 2006; Melacci and Belkin, 2011). The underlying assumption in LapSVM is that the marginal distribution of the data can be represented in a low-dimensional manifold that is representable by a similarity graph. Formally speaking, if the marginal distribution of the data can be supported on a low-dimensional Riemannian manifold, then by exploiting its intrinsic geometry through enforcing a smoothness constraint, one can introduce a preferential bias in the learning process to yield a more accurate model. Thus, by adding a new regularizer term for the smoothness on the manifold, one can expand the framework of supervised learning methods that are fully described by a cost function and regularizers such as SVM and ridge regression to exploit the structure of the data using both the labeled and the unlabeled data. Consequently, the Laplacian SVM solution is defined as

$$f^{*}=\underset{f\in {ℋ}_{K}}{\arg\min}\frac{1}{l}\sum_{i=1}^{l}\gamma_{A}{\left\|f\right\|}_{K}^{2}+\gamma_{I}+\int_{x\in ℳ}{\left\|\nabla_{ℳ}f\right\|}^{2}dP_{X}\left(x\right)$$ (1)

where V is the cost function, $\gamma_{A}$, $\;\gamma_{I}$ are the regularizer coefficients in the so-called ambient and the manifold spaces, respectively, $P_{X}\left(x\right)$ is the marginal distribution of the data, and ${ℋ}_{K}$ is the corresponding reproducing kernel space. Belkin et al. (2006) showed that, under certain conditions, the term corresponding to the manifold regularization can be approximated with $\frac{\gamma_{I}}{N^{2}}f^{T}Lf$, where N is the number of samples, $f={\left[f\left(x_{1}\right),\ldots ,f\left(x_{N}\right)\right]}^{T}$, and L is the Laplacian of the graph underlying the data. As a result, solvers that optimize the supervised SVM problems efficiently can be readily used to find the solution to the semi-supervised LapSVM problem too.

### Restricted Boltzmann Machines

**FIGURE 1:**
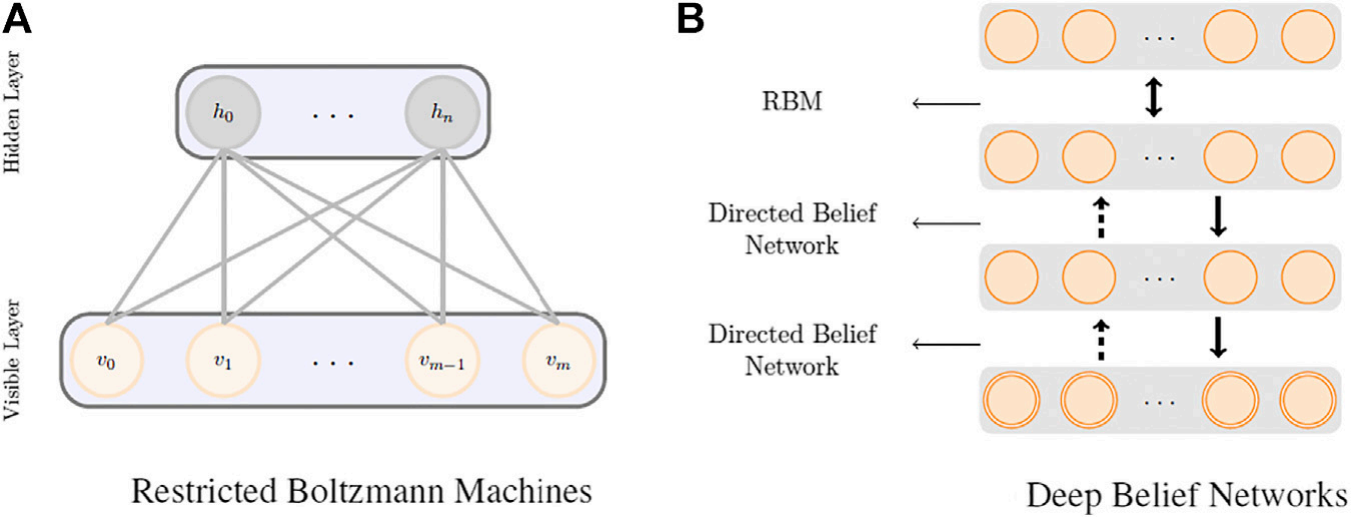
RBM **(A)** and DBN **(B)** model architectures.

RBMs (Hinton and Salakhutdinov, 2006) are the most common building blocks in deep probabilistic models such as DBNs (Goodfellow et al., 2016). These are undirected probabilistic graphical models with a fully bipartite graphical structure (see Figure 1A) that contains a layer of visible units, v, and a layer of latent variables, h. Due to the expressiveness of these models, they have become popular techniques in learning features that are represented by the latent layers. RBMs can also be stacked on top of each other to make deeper architectures. Each unit in an RBM is a binary random variable, and the visible layer of the first RBM in the stack represents the input data. The joint probability distribution in an RBM is modeled as

$$P\left(v,h\right)=\frac{1}{Z}\text{exp}\left(-E\left(v,h\right)\right),$$ (2)

where $E\left(v,h\right)=-b^{T}v-c^{T}h-v^{T}\mathrm{Wh}$ is the energy function, Z is the partition function that normalizes the energy, and *W* is the weight matrix that characterizes the underlying model. Despite the intractable nature of the joint distribution due to the partition function in Eq. 2, $P\left(v,h\right)=\frac{1}{Z}\exp\left(-E\left(v,h\right)\right)$, the conditional distributions are factorial in nature, that is,

$$\begin{array}{c} P\left(h_{j}=1|v\right)=\sigma \left(c_{j}+v^{T}W_{:,j}\right),\;\\ P\left(v_{j}=1\;|h\right)=\sigma \left(b_{j}+h^{T}W_{j,:}\right), \end{array}$$

where σ is the logistic sigmoid function. This makes the overall distribution amenable to sampling, and hence, efficient estimation of the joint probability distribution under the model can be made.

### Deep Belief Networks

The DBN was one of the first attempts that outperformed the state-of-the-art shallow learners in image processing and marks the beginning of the deep learning revolution. Even though this class of deep models do not perform as well as the more advanced deep models when a large body of labeled data is available, they do surprisingly well in circumstances with less data.

Figure 1B shows a schematic representation of a deep belief network. DBNs are generative models formed by stacking several directed belief networks trying to capture causal relations and an RBM layer on the top that acts as an associative model. The joint probability distribution for a DBN with l layers is given by

$$\begin{array}{c} P\left(x=h^{\left(0\right)},h^{\left(1\right)},\ldots ,h^{\left(l\right)}\right)=\left(\overset{l-2}{\underset{k=0}{\prod }}P\left(h^{k}|h^{k+1}\right)\right)P\left(h^{\left(l\right)},h^{\left(l-1\right)}\right),\\ P\left(h^{\left(l\right)},h^{\left(l-1\right)}\right)\propto \exp\left(b^{\left(l\right)}h^{\left(l\right)}+b^{\;{\left(l-1\right)}^{T}}h^{\left(l-1\right)}+h^{{\left(l-1\right)}^{T}}\;Wh^{\left(l\right)}\;\right),\\ P\left(h^{k}|h^{k+1}\right)=\sigma \left(b_{i}^{\left(k\right)}+W_{:,i}^{{\left(k+1\right)}^{T}}h^{\left(k+1\right)}\right)\forall i,k\in 1,\ldots ,l-2, \end{array}$$

where ***b*** ^(*l*)^, ***W*** ^(*l*)^ are the bias vector and the weight vector for the lth layer, respectively.

Thus, DBNs provide multi-layer probabilistic representations of data in an unsupervised way, and as a result, latent representation of the low-level features can be obtained using several levels of abstraction. Training and inference in deep belief nets is not a tractable task. We adopt a heuristic approach called the contrastive divergence (CD-k) proposed by Hinton and Salakhutdinov (2006) to do the training and inference in our model. In summary, this approach begins with training an RBM to maximize $E_{v∼p_{data}}$ and then another RBM to approximately maximize $E_{v∼p_{data}}E_{h^{\left(1\right)}\;∼p^{\left(1\right)}\left(h^{\left(1\right)}|v\right)\;}\log p^{\left(2\right)}\left(h^{\left(1\right)}\right)$, where $p^{\left(1\right)}$and $p^{\left(2\right)}$ are the probability distributions characterized by the first and the second RBMs, respectively. In other words, the second RBM is trained to model the distribution over its input derived from sampling the first RBM. This process can be repeated for as many layers as needed and increases the variational lower bound on the log-likelihood of the data each time a new layer is added. The DBN initializes the weights of multi-layer perceptrons (MLPs), a procedure dubbed as pre-training (Hinton and Salakhutdinov, 2006), to set the stage for the fine-tuning phase in the next step. Specifically, by adding a sigmoid layer on top of a DBN and reusing the generatively trained weights as the initial weights, we can discriminatively train the underlying MLP (Bengio, 2007) via conventional back-propagation–based techniques to converge to a more accurate local optimum. Pre-training differentiates itself from the SSL techniques by finding a proper initial point within the complex search space in an informed way, without modifying the objective function (Erhan, 2010).

### Model Architecture

**FIGURE 2:**
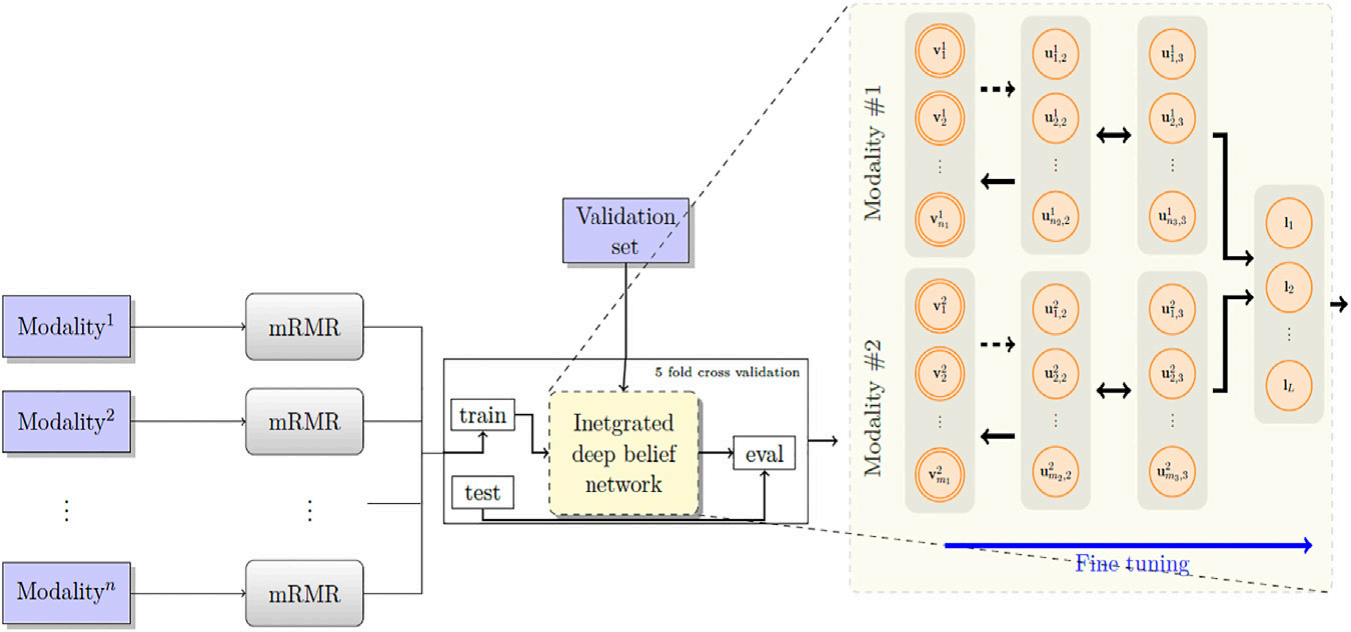
The proposed model RBM. A set of features are first selected for each molecular profile, using mRMR. Then, for each molecular profile, latent features are derived using deep belief nets, which are then fed into a sigmoid layer for downstream prediction.

Figure 2 depicts the architecture of the proposed model. First, the patients’ overall survival statuses are retrieved from the clinical data in TCGA. Patients in the KIRC, HNSC, and NB datasets who at the time of their last follow-up had survived for at least 5, 2.5, and 9 years, respectively, were assigned to the positive survival class. Similarly, patients who did not survive for the corresponding period of time were assigned to the negative (deceased) class, and the rest, i.e., patients whose latest statuses were known to be alive and who yet did not live with their disease long enough to pass the selected threshold, were put into the unlabeled set. Table 1 demonstrates the number of positive, negative, and, unlabeled patients. With each of the datasets, 15% of the labeled samples randomly selected to be a validation dataset. The remaining 85% samples in the labeled set are put into five-folds to conduct a five-fold cross-validation for later analysis of our proposed pipeline. Next, we use mRMR (Peng et al., 2005) to reduce the dimensionality of input modalities so that the uninformative features are removed. mRMR is an incremental search algorithm that looks for a subset of features with the highest relevance to the class labels and lowest redundancy compared to each other. To select the most relevant and yet least redundant probes from the underlying molecular profiles, we discretized the scores pertaining to each probe into three bins, based on its standard deviation across samples (i.e., (−∞,−0.7σ], (−0.7σ, 0.7σ), and [0.7σ,∞)). We then picked the set of top 50 mRMR selected probes (in a non-discretized form) from all modalities combined, excluding the microRNA, as well as the top 20 probes from the microRNA profiles. Subsequently, we computed the per-probe *z*-score of the resulting subset of features before feeding them into our models. It is worth noting that as our DBN-based models are theoretically capable of extracting higher-order informative features from a pool of raw input features, selecting an optimal number of features from the molecular profiles is not a concern here, as long as we choose a proportionate number of input features.

Next, we built a network with two hidden layers (the first layer being a belief net comprising 15 neurons and the second layer is a restricted Boltzmann machine with another 15 neurons). We trained the model corresponding to each modality using the contrastive divergence algorithm with k set to one, i.e., CD-1, used the stochastic gradient descent with a batch size of 25 and a weight decay of 0.001, and continued pre-training for 3,000 epochs to train the network. Subsequently, we augmented our probabilistic DBN model with an additional fully connected sigmoid layer followed by a softmax layer and initialized the weights of the previous layers with those found by the CD-1 algorithm, as this has been shown to be a valuable initialization for such networks. Furthermore, we used our labeled data in the training set to fine-tune the model with a maximum of 500 epochs according to an early-stopping training strategy.

Because cancer has been known to be the outcome of dysregulation of cellular mechanisms in different levels, a single molecular data modality may not adequately explain the sophisticated underlying mechanisms. To account for the interactions, or otherwise correlations between molecular factors with respect to the endpoint we are exploring (which is the risk category of patients), we formed a hybrid model by fusing the intermediate-level features (i.e., features that were generated before the softmax layers) for pairs^2^ of single-modality models and stacked a softmax layer on top of them (see Figure 2). We also explored different model spaces by adding more layers on top of the fusion layer as it theoretically could result in capturing more intricate interactions and hence better performance gains, and we found that such architectures do not bring about further improvements, which can be explained by the limited size of our training sets and the complexity of the task. Finally, we trained the overall model end-to-end, using the cross-entropy loss and the stochastic gradient descent optimizer.

## Results

In this study, we investigate two major questions. First, would a deep classifier help improving the performance of single-modality models in predicting survivals? Second, would the integrated deep belief net outperform the single-modality models? Positive answers to these questions would support the applicability of deep networks in predicting survival and the feasibility of DBNs in utilizing the redundant intermediate features to boost the prediction performance. We compared the performance of the proposed model with two baselines: 1) when we substitute the deep belief parts with the supervised support vector machine (SVM) classifiers and 2) when we use semi-supervised graph-based Laplacian SVMs as a surrogate method. To address the overfitting and underfitting problems due to the inappropriate number of selected input features for the baselines, we use a validation set to choose the best number of features output by the mRMR feature selector. The validation sets are also used to tune baselines’ hyper-parameters in Eq. 1. For the support vector machines, we used the linear kernel as it resulted in the best performance, in which case the performance remains robust with respect to variations in the only model’s hyper-parameter, *C*. We performed a grid search to find an appropriate number of input probes, trying all numbers in the range (Belkin et al., 2006; Alipanahi et al., 2015) with increments of five.^3^ Furthermore, to make an unbiased comparison between the baselines, for SVM, we used the same implementation and solver as used in the LapSVM method. For our LapSVM model, on the contrary, we performed a grid search on the set of model’s hyper-parameters^4^ in addition to the size of selected input probes. We found that the degree of 1, for the Laplacian graph, and the sigma of 3.0 for the model’s RBF kernel remain the same across all the sets of parameter configurations. For the extrinsic and intrinsic regularization parameters, we searched the logarithmic search spaces [*1e*−*2,1e2*] and [*0,1e2*], respectively.

### Single-Modality Models

**FIGURE 3:**
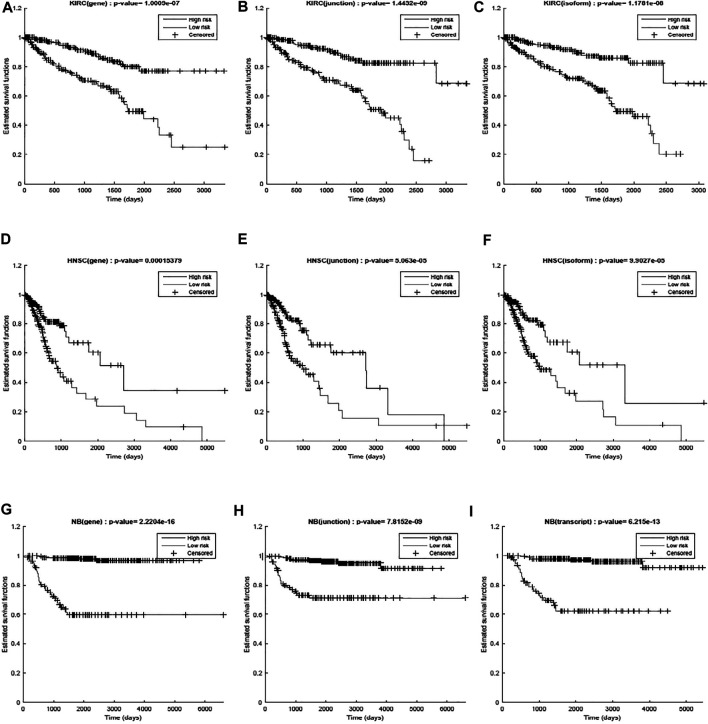
Kaplan–Meier curves and the log-rank *p*-values for the predictions made by the proposed work for different diseases (KIRC, HNSC, and NB) per different modalities (gene, junction, and isoform). **(A)** RNA-seq gene, **(B)** RNA-seq junction, and **(C)** RNA-seq isoform for kidney cancer; **(D)** RNA-seq gene, **(E)** RNA-seq junction, and **(F)** RNA-seq isoform for head and neck cancer; **(G)** RNA-seq gene, **(H)** RNA-seq junction, and **(I)** RNA-seq isoform for neuroblastoma. The curves show a clear separation between the two predicted groups.

**FIGURE 4:**
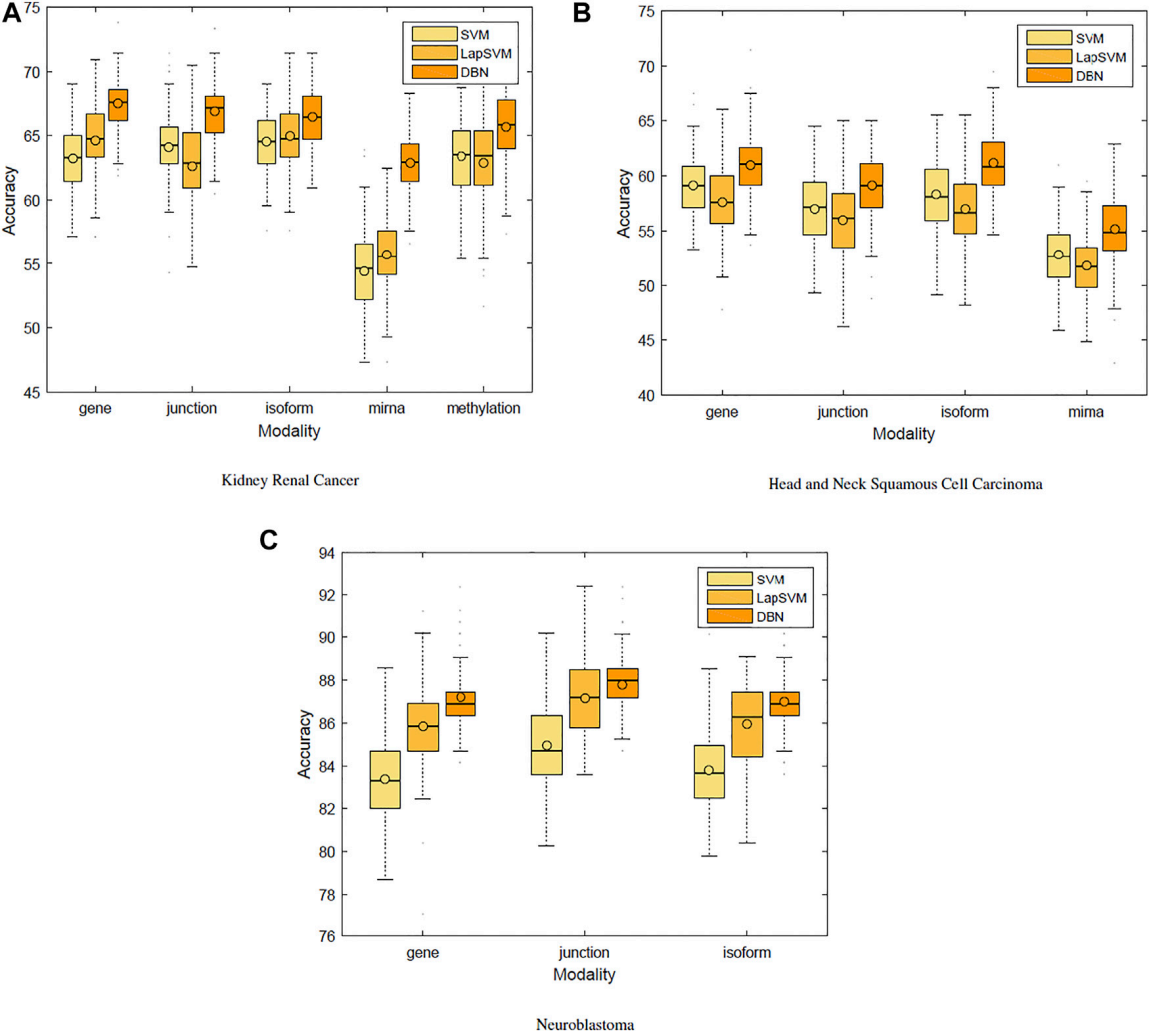
Benchmarking support vector machines, Laplacian SVM, and deep belief networks.

We first used only one modality as input (hence the single-modality model) to show how deep belief networks can be trained on relatively small cancer datasets to predict the survival. Figure 3 depicts the Kaplan–Meier (KM) curves along with the corresponding log-rank *p*-values for the predictions made by our deep predictor for the three RNA-seq modalities (i.e., gene, junction, and isoform). For each labeled sample, we trained the model once on all but that sample and made a prediction on it, repeated this process for all samples, and plotted the KM curves for the combined predictions. According to the figure, our approach produces meaningful clusters of high-risk and low-risk patients. Furthermore, we benchmarked the proposed predictor against the SVM and the Laplacian SVM (LapSVM). Specifically, we randomly split cancer datasets into the train, test, and validation sets 100 times and subsequently trained deep models and baselines once for each input modality listed in Table 1. Figure 4 illustrates the boxplots of accuracies achieved during this experiment. According to this figure, the trends observed in the prognostic power of individual molecular datasets correlate and strongly depend on the cancer type. Furthermore, the DBN is doing consistently better than baseline methods on average. Importantly, this improvement comes with a tighter confidence interval, as illustrated in Table 2. Interestingly, despite the relative success of the semi-supervised LapSVM in leveraging the unlabeled data, it is unable to surpass the supervised SVM for some input modalities. This is because semi-supervised methods need a significant amount of unlabeled data to learn the distribution of input space efficiently, which does not hold for most cancer datasets. DBN models, however, are more immune to this shortcoming as evidenced by the results presented here.

**TABLE 2:** Mean (SD) of accuracies for 100 randomly initialized runs. The DBN has the smallest variance for the majority of datasets/modalities.

		Modality				
		Gene	Junction	Isoform	miRNA	Methylation
Disease	KIRC	63.27 (2.64)	65.13 (2.80)	64.52 (2.53)	54.43 (3.38)	63.39 (3.08)
		64.62 (2.76)	62.65 (3.23)	65.0 (2.99)	55.71 (2.79)	62.88 (3.59)
		67.48 (**2.19**)	66.97 (**2.50**)	66.49 (**2.24**)	62.9 (**2.47**)	65.71 (**3.0**)
	HNSC	59.15 (2.78)	56.94 (3.57)	58.33 (3.12)	54.43 (3.38)	
		57.61 (**3.13**)	55.99 (3.79)	56.96 (3.19)	55.72 (**2.79**)	
		61.04 (3.2)	59.22 (**3.1**)	61.14 (**2.85**)	61.14 (2.85)	
	NB	83.39 (1.88)	84.94 (2.12)	83.81 (1.96)		
		85.86 (2.04)	87.15 (1.86)	85.96 (1.97)		
		87.18 (**1.6**)	87.78 (**1.49**)	86.97 (**1.49**)		

### The Multi-Modality Pipeline

Cancer is known to be a disease associated with dysregulation of cellular mechanisms in different levels. Hence, no single molecular modality is sufficient to predict cancer-related endpoints, such as the survival (Chin and Gray, 2008). Therefore, changes in biological pathways may be captured more accurately if different modalities are integrated together seamlessly. Figure 4 suggests that the most effective molecular modality regarding the prediction performance is different across different cancer types. For instance, for the kidney cancer dataset, the model trained over the RNA-seq: gene modality results in the most accurate predictor, whereas in case of neuroblastoma, RNA-seq: junction modality leads to the most accurate model. Ideally, we would like to have an integrated pipeline that is more accurate than each of the single modality models individually. Our goal in this section is to examine whether adding another molecular data modality can provide more prognostic power given the proposed integration paradigm through an additional sigmoid layer that is stacked on top of the RBM layer.

**FIGURE 5:**
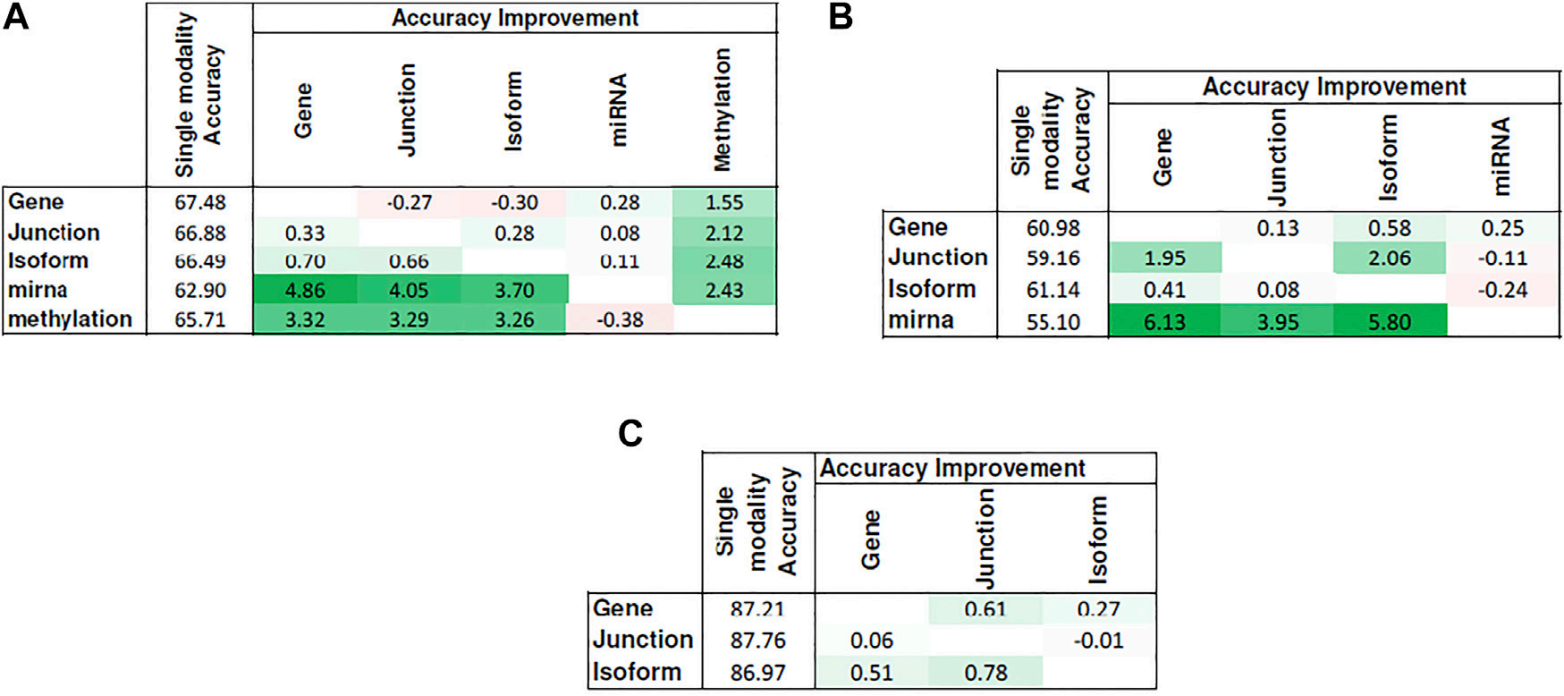
Improvement achieved after integrating pairs of modalities. Columns show the additional modality added to the single-modality model (denoted by rows). Cell values show the difference in accuracy between the integrated model and the single-modality model. Tables **(A)–(C)** correspond to KIRC, HNSC, and NB, respectively.

Figure 5 shows the heat map of accuracy improvements when pairs of different input modalities are combined according to the final pipeline design. Our results suggest that integration of latent features generated by deep belief networks from different modalities leads to improvements for majority of the cases. This improvement, however, is not significant in case of integration of two RNA-seq modalities. This is because they are different representations of the same source of information and combining them will add little additional predictive value. On the contrary, combining data of different molecular levels can lead to more substantial improvements as is the case with the integration of methylation/miRNA and the RNA-seq data modality. Note that, for the HNSC dataset, miRNA does not provide additional improvement, which is in agreement with recent findings that miRNA is not directly related to the disease prognosis (Hess et al., 2019), as also indicated by its poor prediction accuracy^5^ in Figure 4. Hence, its addition does not result in any improvement. Interestingly, despite the large difference in the model’s performance when trained on miRNA vs. other profiles, the drop in performance of the integrated model is negligible, suggesting that the integrated model can offer robustness as well as synergistic gains in performance.

## Conclusion

In this study, we developed a deep learning–based pipeline to predict cancer survival. Because of the unsupervised nature of the pre-training stage, we were able to leverage the unlabeled and censored data to arrive at a better initialization of the model parameters. Such an initialization is a critical step that drives the final fine-tuned model to a more biologically relevant point in the parameter space particularly when the number of layers in the model increases. Our results showed that the proposed model architecture can indeed achieve this goal by successfully exploiting the information that is available in such data and subsequently integrating derived features from different molecular profiles. This is corroborated by the fact that our trained models consistently outperformed the semi-supervised baseline. Moreover, we showed that the most informative data modality can be different across different cancer types, which justifies the need for an integrated decision support system that has the ability to generate synergistic improvements from multiple available modalities. It is worth noting that the focus and scope of this study was on presenting the merits of deep models for extracting informative features from molecular profiles of cancer tumors in an integrated manner. Needless to say that including more modalities such as clinical and proteomic data can enhance the prediction performance even further, as shown in other studies (Liu et al., 2014; Yuan et al., 2014), and can be considered a future work for a more comprehensive decision support system. Another direction that requires further exploration and attention is to evaluate the robustness of such models in light of data scarcity and data variation. The presented approach was an effort to address this challenge by exploiting unlabeled data; however, an important question would be how models trained on data from one study are generalizable and applicable to the data acquired for the same disease but from another study. Finally, it is desirable to know the strengths and limitations of deep belief networks with other pre-training frameworks used for training deep models, such as the variational auto-encoders (An and Cho, 2015) and the more recent contrastive learning (Falcon and Cho, 2020) framework.

Despite all their success in extracting informative latent features from data, deep models are considered black boxes that learn by simple associations and co-occurrences (Mamoshina et al., 2016). This obviates the need for human intervention to generate hand-crafted features or to use the expert knowledge but comes at cost of lacking transparency and interpretability in such models. Making deep interpretable models is currently an active research that has caught attention of researches in the machine learning community and is another dimension where this work can be expanded as a future work.

## Data Availability

Publicly available datasets were analyzed in this study. These data can be found at https://www.cancer.gov/about-nci/organization/ccg/research/structural-genomics/tcga

## Footnotes

1 Even though DBNs partially alleviate data scarcity in training, when the dataset is too small, they cause data overfitting.

2 We tried the fusion of more than two data modalities as well; however, this led to model overfitting and a drop in accuracy, due to the small size of datasets. We hypothesize that adding more modalities can lead to a synergistic boost in the prediction performance if more data become available.

3 We observed consistent performance drops due to model overfitting if more than 35 probes are selected.

4 Sigma for the RBF kernel, the number of neighbors, the extrinsic and intrinsic regularization parameters, and the power (degree) of the graph Laplacian to use as the graph regularizer.

5 The low accuracies reported for HNSC can be attributed to the biological variability of samples, as this cancer type includes cancer of the oral cavity, pharynx, larynx, nasal cavity, and salivary glands.
